# Attention-Based Deep Neural Networks for Detection of Cancerous and Precancerous Esophagus Tissue on Histopathological Slides

**DOI:** 10.1001/jamanetworkopen.2019.14645

**Published:** 2019-11-06

**Authors:** Naofumi Tomita, Behnaz Abdollahi, Jason Wei, Bing Ren, Arief Suriawinata, Saeed Hassanpour

**Affiliations:** 1 Department of Biomedical Data Science, Geisel School of Medicine, Dartmouth College, Hanover, New Hampshire; 2 Department of Computer Science, Dartmouth College, Hanover, New Hampshire; 3 Department of Pathology and Laboratory Medicine, Dartmouth-Hitchcock Medical Center, Lebanon, New Hampshire; 4 Department of Epidemiology, Geisel School of Medicine, Dartmouth College, Hanover, New Hampshire

## Abstract

**Importance:**

Deep learning–based methods, such as the sliding window approach for cropped-image classification and heuristic aggregation for whole-slide inference, for analyzing histological patterns in high-resolution microscopy images have shown promising results. These approaches, however, require a laborious annotation process and are fragmented.

**Objective:**

To evaluate a novel deep learning method that uses tissue-level annotations for high-resolution histological image analysis for Barrett esophagus (BE) and esophageal adenocarcinoma detection.

**Design, Setting, and Participants:**

This diagnostic study collected deidentified high-resolution histological images (N = 379) for training a new model composed of a convolutional neural network and a grid-based attention network. Histological images of patients who underwent endoscopic esophagus and gastroesophageal junction mucosal biopsy between January 1, 2016, and December 31, 2018, at Dartmouth-Hitchcock Medical Center (Lebanon, New Hampshire) were collected.

**Main Outcomes and Measures:**

The model was evaluated on an independent testing set of 123 histological images with 4 classes: normal, BE-no-dysplasia, BE-with-dysplasia, and adenocarcinoma. Performance of this model was measured and compared with that of the current state-of-the-art sliding window approach using the following standard machine learning metrics: accuracy, recall, precision, and F1 score.

**Results:**

Of the independent testing set of 123 histological images, 30 (24.4%) were in the BE-no-dysplasia class, 14 (11.4%) in the BE-with-dysplasia class, 21 (17.1%) in the adenocarcinoma class, and 58 (47.2%) in the normal class. Classification accuracies of the proposed model were 0.85 (95% CI, 0.81-0.90) for the BE-no-dysplasia class, 0.89 (95% CI, 0.84-0.92) for the BE-with-dysplasia class, and 0.88 (95% CI, 0.84-0.92) for the adenocarcinoma class. The proposed model achieved a mean accuracy of 0.83 (95% CI, 0.80-0.86) and marginally outperformed the sliding window approach on the same testing set. The F1 scores of the attention-based model were at least 8% higher for each class compared with the sliding window approach: 0.68 (95% CI, 0.61-0.75) vs 0.61 (95% CI, 0.53-0.68) for the normal class, 0.72 (95% CI, 0.63-0.80) vs 0.58 (95% CI, 0.45-0.69) for the BE-no-dysplasia class, 0.30 (95% CI, 0.11-0.48) vs 0.22 (95% CI, 0.11-0.33) for the BE-with-dysplasia class, and 0.67 (95% CI, 0.54-0.77) vs 0.58 (95% CI, 0.44-0.70) for the adenocarcinoma class. However, this outperformance was not statistically significant.

**Conclusions and Relevance:**

Results of this study suggest that the proposed attention-based deep neural network framework for BE and esophageal adenocarcinoma detection is important because it is based solely on tissue-level annotations, unlike existing methods that are based on regions of interest. This new model is expected to open avenues for applying deep learning to digital pathology.

## Key Points

**Question:**

Can deep learning approaches accurately identify cancerous and precancerous esophagus tissue on microscopy images without training on region-of-interest annotations?

**Findings:**

In this diagnostic study of 123 histological images, a novel deep learning method, trainable without region-of-interest annotations, analyzed Barrett esophagus and esophageal adenocarcinoma whole-slide images and achieved a mean accuracy of 0.83 in classifying the test images. These results were comparable with or better than the performance from the current state-of-the-art sliding window approach, which was trained with regions of interest.

**Meaning:**

Findings of this study suggest that the proposed model eliminates the annotation bottleneck in developing deep learning models for whole-slide image analysis and is a generalizable approach to characterizing histological patterns on high-resolution microscopy images.

## Teaser

This diagnostic study describes a novel attention-based deep neural network framework for classifying microscopy images to identify Barrett esophagus and esophageal adenocarcinoma.

## Introduction

Barrett esophagus (BE) is a transformation of the normal squamous epithelium of the esophagus into metaplastic columnar epithelium.^1^ Barrett esophagus is important because it predisposes patients to the increased risk of adenocarcinoma of the esophagus and gastroesophageal junction.^2,3^ Compared with the general population, patients with BE have a 30 to 125 times higher risk of cancer.^4^ The mean 5-year survival rate for esophageal adenocarcinoma (EAC) is less than 15% in the United States.^5^ Furthermore, the incidence of EAC in the United States increased dramatically over 3 decades.^6,7,8,9,10^ Histological diagnosis of BE requires the identification of metaplastic columnar epithelium with goblet cells (ie, intestinal metaplasia).^11^ Evaluating the development of the premalignant and malignant neoplasm in BE shows a moderate interobserver variability, with a mean κ coefficient of less than 0.50 even among subspecialized gastrointestinal pathologists.^12^

In digital pathology, tissue slides are scanned as high-resolution images. High resolution is necessary because each slide contains thousands of cells, for which the cellular structures must be visible to allow the identification of regions of the tissue with diseases or lesions. The size of lesions is often relatively small, and most of the tissue areas in a given slide are normal. Even for highly trained pathologists, localizing the decisive regions of interest (ROIs) containing lesions for the classification of the whole slide is time-consuming and prone to miss an ROI.

In recent years, deep learning has made considerable advances in classifying microscopy images. The most common approach in this domain involves a sliding window model for cropped-image classification, followed by statistical methods of aggregation for whole-slide inference.^13,14,15,16,17,18,19,20,21,22,23^ In the sliding window approach, pathologists annotate bounding boxes (ie, ROIs) on whole slides to train a classifier on small cropped images, typically in sizes ranging from 200 × 200 pixels to 500 × 500 pixels. For evaluating a whole slide, this cropped-image classifier is applied to extracted windows from the image, and then a heuristic, often developed in conjunction with a domain-expert pathologist, is used to determine how the distribution of cropped-image classification scores translates into a whole-slide diagnosis.

The sliding window approach has several limitations, however. First, given that cropped-image classifiers are needed, all images in the training set must be annotated by pathologists with bounding boxes around each ROI. Second, developing a heuristic for aggregating cropped-image classifications, which requires pathologist insight, is dependent on the nature of the classification task and is not widely scalable. Third, cropped images are classified independently of their neighbors, and whole-slide classification does not consider correlations between neighboring windows. To overcome these limitations in this study, we developed an attention mechanism that mines the ROI from high-resolution slides without explicit supervision.

Our work was inspired by attention models applied to regular-image analysis tasks, especially image captioning.^24,25^ Attention mechanisms are described as a part of the prediction module that sequentially selects subsets of input to be processed.^24^ Although this definition is not applicable to nonsequential tasks, the essence of attention mechanisms can be restructured for neural networks to generate a dynamic representation of features by weighting them to capture a holistic context of input. Unlike hard attention, in which an ROI is selected by a stochastic sampling process, soft attention generates a nondiscrete attention map that pays fractional attention to each region and produces better gradient flow and thus is easier to optimize. Recent advancement of soft attention enabled end-to-end training on convolutional neural network models.^26,27,28,29^ For example, spatial transformer networks capture high-level information from inputs to derive affine transformation parameters, which are subsequently applied to spatial invariant input for a convolutional neural network.^29^ For semantic segmentation tasks, the attention mechanism is applied to learn multiscale features.^26^ Residual attention networks use soft attention masks to extract features in different granularities.^28^

For analyzing images in detail, a top-down, recurrent attention, convolutional neural network has been proposed.^27^ To put our work into perspective, that proposed model is based on the soft attention mechanism in feature space but is designed for the classification of high-resolution images that are not typically encountered in the field of computer vision. The attention mechanism has several applications in the medical domain, such as using soft attention to generate masks around lesion areas on computed tomography images^30^ and using recurrent attention models fused with reinforcement learning to locate lung nodules^31^ or enlarged hearts^32^ in chest radiography images. In pathology, recorded navigation of pathologists has been used as attention maps to detect carcinoma.^33^ Soft attention has been deployed in 2 parallel networks for the classification of thorax disease.^30^ Although we drew inspiration from this earlier work, our proposed attention-based model is different in that it provides a novel framework to directly reuse extracted features in a single attention network.

In this study, we developed a model that uses a convolutional attention-based mechanism to classify microscopy images. This attention-based model has 3 major advantages over the existing sliding window method. First, our model dynamically identifies ROIs in a high-resolution image and makes a whole-slide classification based on the analysis of only selected regions. This process is analogous to how pathologists examine slides under the microscope. Second, our proposed model is trainable end to end with only tissue-level labels. All components of the model are optimized through backpropagation. Unlike the sliding window approach, the model does not need bounding box annotations for ROIs or pathologist insight for heuristic development. Third, our model has a flexible architecture with regard to input size for images. Inspired by fully convolutional network philosophy,^34^ the model’s grid-based attention module uses a 3-dimensional (3-D) convolution operation that does not require a fixed-size input grid. The input size can be any rectangular shape that fits in the memory of graphic processing units, which all modern deep learning frameworks use to accelerate computations.

## Methods

### Data Set

For this diagnostic study, whole-slide images were collected from patients who underwent endoscopic esophagus and gastroesophageal junction mucosal biopsy between January 1, 2016, and December 31, 2018, at Dartmouth-Hitchcock Medical Center, a tertiary academic medical center in Lebanon, New Hampshire. The use of data collected for this study was approved by the Dartmouth Institutional Review Board, which waived the requirement of informed consent as the collected data were deidentified. The study is in compliance with the Declaration of Helsinki on Ethical Principles for Medical Research Involving Human Subjects.^35^ In addition, the study followed the Standards for Reporting of Diagnostic Accuracy (STARD) reporting guidelines.^36^

A scanner (Aperio AT2; Leica Biosystems Inc) was used to digitize hematoxylin-eosin–stained whole-slide images at 20× magnification. Scanning with 20× magnification is routinely performed in the clinical workflow for faster scanning throughput and efficient file size. We had a total of 180 whole-slide images, of which 116 (64.4%) were used as the training set and 64 (35.6%) were used as the testing set. Of the training set, 23 whole-slide images (19.8%) were reserved for validation. These whole-slide images can cover multiple pieces of tissue. Therefore, the whole-slide images were separated into 379 high-resolution images later in the preprocessing step, with each image covering a single piece of tissue.

To determine labels for whole-slide images and to train the existing state-of-the-art sliding window approach as the baseline, 2 of our expert pathologists from the Department of Pathology and Laboratory Medicine at Dartmouth-Hitchcock Medical Center (A.S., B.R.) annotated bounding boxes around lesions in these images (eMethods 1 in the Supplement). We considered these labels as the reference standard, as any disagreements in annotation were resolved through further discussion among our senior domain-expert pathologist annotators. These bounding boxes were not needed in training the proposed attention-based model.

This study used categories of esophageal dysplasia and carcinoma based on the Vienna classification system.^37^ The normal class included normal squamous epithelium, normal squamous and columnar junctional epithelium, and normal columnar epithelium. Barrett esophagus negative for dysplasia was included in the BE-no-dysplasia class. Barrett esophagus is defined by columnar epithelium with goblet cells (intestinal metaplasia) and preservation of orderly glandular architecture of the columnar epithelium with surface maturation. The BE-with-dysplasia class included low-grade dysplasia (noninvasive low-grade neoplasia) and high-grade dysplasia (noninvasive high-grade neoplasia). Columnar epithelium with low-grade dysplasia is characterized by nuclear pseudostratification, mild to moderate nuclear hyperchromasia and irregularity, and the cytologic atypia extending to the surface epithelium. High-grade dysplasia demonstrated marked cytologic atypia, including loss of polarity, severe nuclear enlargement and hyperchromasia, numerous mitotic figures, and architectural abnormalities such as lateral budding, branching, and villous formation as well as variation in the size and shape of crypts.

In contrast to the Vienna classification system, we merged BE with low-grade dysplasia and high-grade classes into 1 class owing to the low number of collected samples for each class. The adenocarcinoma class included invasive carcinoma (intramucosal carcinoma and submucosal carcinoma and beyond) and high-grade dysplasia suggestive of invasive carcinoma. Cases in the adenocarcinoma class may present the following features: single-cell infiltration, sharply angulated glands, small glands in a back-to-back pattern, confluent glands, cribriform or solid growth, ulceration occurring within high-grade dysplasia, dilated dysplastic glands with necrotic debris, or dysplastic glands undermining squamous epithelium.

### Two-Step Method and Testing

**Figure 1.**
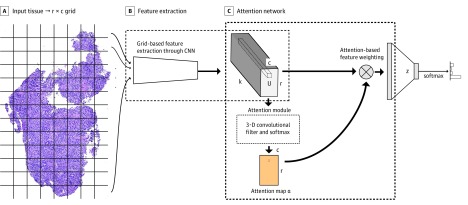
Overview of Proposed Attention-Based Model A, An input image is divided into r × c grid cells (dividing lines are shown only for visualization). B, Features extracted from each grid cell build a grid-based feature map tensor U. C, Learnable 3-dimensional convolutional filters of size k × d × d (where d denotes the height and width of the convolutional filters) are applied on U feature map to generate an attention map α, which operates as the weights for an affine combination of feature vectors in U. The α represents a 2-dimensional attention map whose size is r in height and c in width; CNN, convolutional neural network; r and c, the number of rows and columns of input tissue grid; U, a tensor of features extracted from each grid cell, and its size is r in height, c in width, and k in depth; and z, a vector of features representing a whole-input image.

The proposed attention-based model has 2 steps, which are shown in Figure 1. The first step is the extraction of grid-based features from the high-resolution image, at which point each grid cell in the whole slide is analyzed to generate a feature map (Figure 1A and B). The second step is the application of the attention mechanism on the extracted features for slide classification (Figure 1C). The feature extractor is jointly optimized across all the grid cells with the attention module in an end-to-end fashion. In the end-to-end training pipeline, the cross-entropy loss over all classes is computed on class predictions. The loss is backpropagated to optimize all parameters in the network without any manual adjustment for attention modules. The model does not need bounding box annotations around ROIs, and all optimization is done to only the labels at the tissue level. Further details of the model architecture of the grid-based feature extraction and attention-based classification are provided in eMethods 2 in the Supplement.

To evaluate the attention-based classification model for high-resolution microscopy images, we applied the steps to high-resolution scanned slides of tissues endoscopically removed from patients who were at risk for esophageal cancer. We compared the performance results of the proposed model with those of the state-of-the-art sliding window approach.^22^

For preprocessing, we removed the white background from the slides and extracted only regions of the images that contained tissue. eFigure 1A in the Supplement shows a typical whole-slide image from the data set. These whole-slide images can cover multiple pieces of tissue, so we separated them into subimages with each covering only a single piece of tissue. The median (interquartile range) width of the tissues was 4500 (3000-6500) pixels and the median (interquartile range) height was 5500 (4000-7500) pixels. Every tissue image was given an overall label based on the labels of its lesions. If multiple lesions with different classes were present, we used the class with the highest risk as the corresponding label, as that lesion would have the highest implication clinically. If no abnormal lesions were found in an image, it was assigned to the normal class. After this preprocessing step, each image was assigned to 1 of 4 classes: normal, BE-no-dysplasia, BE-with-dysplasia, and adenocarcinoma (eFigure 1B in the Supplement).

The data set included 379 images after preprocessing. One-third of the data set was reserved for testing. To avoid possible data leakage, we placed all tissues extracted from 1 whole-slide image into the same set of images when the training and testing sets were split. The Table summarizes the results of the testing set.

**Table.** Classification Results for the Testing Set^a^

Metric	Sliding Window Approach Performance (95% CI)^b^	Attention-Based Model Performance (95% CI)
Normal class		
Accuracy	0.63 (0.56-0.69)	0.70 (0.64-0.76)
Recall	0.62 (0.53-0.71)	0.69 (0.61-0.77)
Precision	0.60 (0.51-0.69)	0.68 (0.59-0.76)
Specificity	0.63 (0.57-0.72)	0.71 (0.62-0.79)
F1 score	0.61 (0.53-0.68)	0.68 (0.61-0.75)
BE-no-dysplasia class		
Accuracy	0.85 (0.80-0.89)	0.85 (0.81-0.90)
Recall	0.43 (0.31-0.56)	0.77 (0.66-0.87)
Precision	0.87 (0.73-0.97)	0.68 (0.57-0.78)
Specificity	0.98 (0.95-1.00)	0.88 (0.83-0.93)
F1 score	0.58 (0.45-0.69)	0.72 (0.63-0.80)
BE-with-dysplasia class		
Accuracy	0.72 (0.66-0.77)	0.89 (0.84-0.92)
Recall	0.36 (0.18-0.54)	0.21 (0.07-0.38)
Precision	0.16 (0.08-0.26)	0.50 (0.20-0.80)
Specificity	0.76 (0.70-0.82)	0.97 (0.94-0.99)
F1 score	0.22 (0.11-0.33)	0.30 (0.11-0.48)
Adenocarcinoma class		
Accuracy	0.87 (0.83-0.91)	0.88 (0.84-0.92)
Recall	0.52 (0.37-0.68)	0.71 (0.57-0.85)
Precision	0.65 (0.48-0.80)	0.63 (0.49-0.76)
Specificity	0.94 (0.90-0.98)	0.91 (0.87-0.95)
F1 score	0.58 (0.44-0.70)	0.67 (0.54-0.77)
Mean		
Accuracy	0.76 (0.73-0.80)	0.83 (0.80-0.86)
Recall	0.48 (0.41-0.56)	0.60 (0.53-0.66)
Precision	0.57 (0.51-0.63)	0.62 (0.53-0.71)
F1 score	0.50 (0.43-0.56)	0.59 (0.52-0.66)

Abbreviation: BE, Barrett esophagus.

^a^ The proposed attention-based model's performance was assessed on the basis of accuracy, recall, precision, specificity, and F1 score. Results were rounded to 2 decimal places. The model outperformed the sliding window baseline in both accuracy and F1 score for all classes.

^b^ The sliding window approach is explained in Wei et al.^22^

### Sliding Window Approach as Baseline

To compare the proposed model with previous methods for high-resolution image analysis, we implemented the current state-of-the-art sliding window approach^22^ as a baseline. For this method, we used the annotated bounding box labels to generate small, cropped images of 224 × 224 pixel size for training a cropped-image classifier. For preprocessing, we normalized the color channels and performed standard data augmentation, including color jittering, random flips, and rotations. For training, we initialized ResNet-18 with MSRA (Microsoft Research Asia) initialization.^38^ We optimized the model with a cross-entropy loss function for 100 epochs, using standard weight regularization techniques and learning rate decay. We trained the cropped-image classifier to predict the class of any given window on a high-resolution image. For whole-slide inference, we performed a grid search of the validation set for optimal thresholds to filter noise. Then, our 2 pathologists (A.S., B.R.) were consulted to develop heuristics for aggregating cropped-image predictions. We chose the thresholds and heuristics that performed the best on the validation set and applied those to the whole-slide images in the testing set.

### Attention-Based Model

We implemented the attention-based model as described. Given the size of features extracted from the ResNet-18 model, we used 512 × 3 × 3, 3-D convolutional filters in the attention module, with implicit zero padding of 0 for depth, 1 for height, and 1 for width dimensions. We used 64 of these filters to increase the robustness of the attention module, as patterns in the feature space are likely too complex to be recognized and attended by a single filter. To avoid overfitting and encourage each filter to capture different patterns, we regularized the attention module by applying dropout^39^ with *P* = .50 after concatenating all of the feature vectors. We initialized the entire network with MSRA initialization for convolutional filters,^38^ unit weight and zero bias for batch normalizations,^40^ and Glorot initialization for fully connected layers.^41^ Only the cross-entropy loss against class labels was used in training. Other information, such as the location of bounding boxes, was not given to the network as guidance to optimal attention maps. The model identified such ROIs automatically.

We initialized the feature extraction network with weights pretrained on the ImageNet data set.^42^ Input for the network was extracted grid cells of 492 × 492 pixels that were resized to 224 × 224 pixels. We normalized the input values by the mean (SD) of pixel values computed over all tissues in the training set. In training, the last fully connected layer of the network was removed, and all residual blocks except for the last one were frozen, serving as a regularization mechanism.

We trained the entire network on large, high-resolution images. For data augmentation, we applied random rotation and random scaling, with a scaling factor between 0.8 and 1.2 during training. We used the Adam optimizer with an initial learning rate of 1 × 10^−3^, decaying by 0.95 after each epoch, and reset the learning rate to 1 × 10^−4^ every 50 epochs in a total of 200 epochs, similar to the cyclical learning rate.^43,44^ We set the mini-batch size to 2 to maximize the use of memory on the graphic processing unit (Nvidia Titan Xp; NVIDIA Corporation). The model was implemented in PyTorch.^45^ At testing, the network took a mean 0.34 seconds to analyze a high-resolution image.

### Statistical Analysis

Data were analyzed in October 2018. For quantitative evaluation, 4 standard metrics were used for classification under a 1-vs-rest strategy: accuracy, recall, precision, and F1 score. To estimate 95% CIs, bootstrapping was used for all metrics. The 2-tailed McNemar-Bowker test was used, and α = .05 was considered statistically significant. Statistical analysis was carried out with SciPy, version 1.0.0 (SciPy developers).

## Results

The data set contained a total of 379 histological images, of which 195 (51.5%) were in the normal class, 80 (21.1%) were in the BE-no-dysplasia class, 46 (12.1%) were in the BE-with-dysplasia class, and 58 (15.3%) were in the adenocarcinoma class. Of the independent testing set of 123 images, 58 (47.2%) normal, 30 (24.4%) BE-no-dysplasia, 14 (11.4%) BE-with-dysplasia, and 21 (17.1%) adenocarcinoma images were used to evaluate trained models and to analyze the classification performance from both quantitative and qualitative aspects. The eTable in the Supplement provides a detailed description of the data set.

The classification results on the testing set are summarized in the Table. Compared with the sliding window baseline, the proposed model achieved better accuracy and F1 score in all classes. Especially for F1 score, which is the harmonic mean of precision and recall, the attention-based model outperformed the sliding window approach by at least 8% for each class: 0.68 (95% CI, 0.61-0.75) vs 0.61 (95% CI, 0.53-0.68) for the normal class, 0.72 (95% CI, 0.63-0.80) vs 0.58 (95% CI, 0.45-0.69) for the BE-no-dysplasia class, 0.30 (95% CI, 0.11-0.48) vs 0.22 (95% CI, 0.11-0.33) for the BE-with-dysplasia class, and 0.67 (95% CI, 0.54-0.77) vs 0.58 (95% CI, 0.44-0.70) for the adenocarcinoma class. However, this outperformance was not statistically significant at the α = .05 level with the McNemar-Bowker test.

Results of the attention-based model had the following sensitivities for recall: 0.69 (95% CI, 0.61-0.77) for the normal class, 0.77 (95% CI, 0.66-0.87) for the BE-no-dysplasia class, 0.21 (95% CI, 0.07-0.38) for the BE-with-dysplasia class, and 0.71 (95% CI, 0.57-0.85) for the adenocarcinoma class. The specificities under this model were as follows: 0.71 (95% CI, 0.62-0.79) for the normal class, 0.88 (95% CI, 0.83-0.93) for the BE-no-dysplasia class, 0.97 (95% CI, 0.94-0.99) for the BE-with-dysplasia class, and 0.91 (95% CI, 0.87-0.95) for the adenocarcinoma class. Classification accuracies of the proposed model were 0.85 (95% CI, 0.81-0.90) for the BE-no-dysplasia class, 0.89 (95% CI, 0.84-0.92) for the BE-with-dysplasia class, and 0.88 (95% CI, 0.84-0.92) for the adenocarcinoma class. The proposed model achieved a mean accuracy of 0.83 (95% CI, 0.80-0.86).

**Figure 2.**
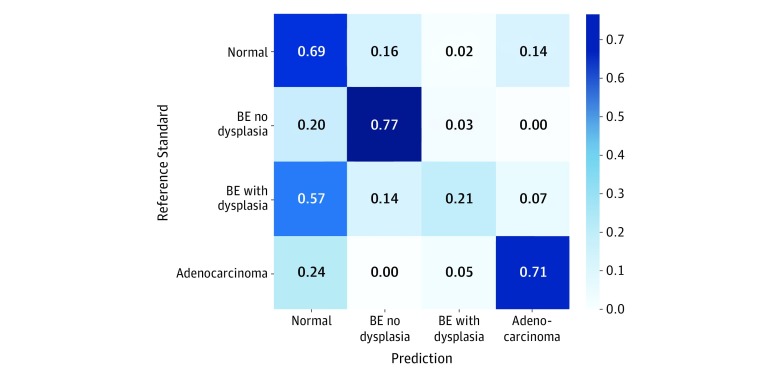
Confusion Matrix for Pathologist Diagnoses and Model Predictions The confusion matrix for different histological classes related to esophageal cancer compares the classification agreement of the attention-based model with pathologist consensus. BE indicates Barrett esophagus.

**Figure 3.**
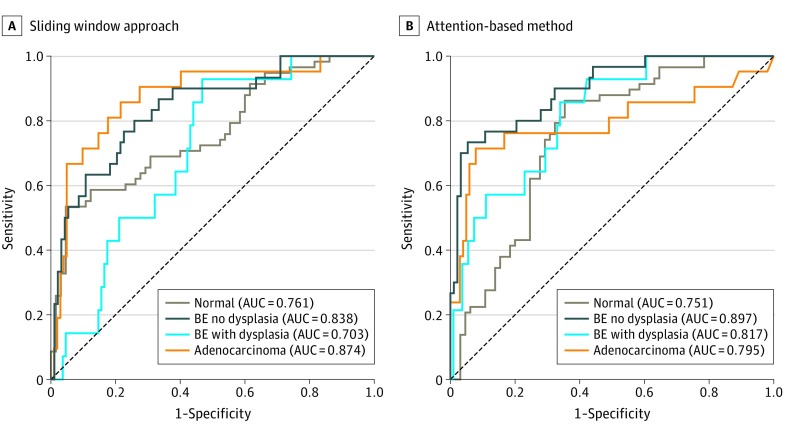
Performance Curves for the Sliding Window Approach and the Attention-Based Model Receiver operating characteristic curves for the sliding window approach (A) and the proposed attention-based method (B) show the true-positive rate (y-axis) and the false-positive rate (x-axis) at various decision threshold levels for each diagnostic class. AUC indicates area under the receiver operating characteristic curve; BE, Barrett esophagus.

Our quantitative analysis showed the proposed model’s good performance as follows: 0.68 (95% CI, 0.61-0.75) for the normal class, 0.72 (95% CI, 0.63-0.80) for the BE-no-dysplasia class, and 0.67 (95% CI, 0.54-0.77) for the adenocarcinoma class. Both the attention-based and the sliding window models, however, did not perform as well in identifying the images of BE-with-dysplasia class: 0.30 (95% CI, 0.11-0.48) for the attention-based model and 0.22 (95% CI, 0.11-0.33) for the sliding window approach. As shown in the confusion matrix in Figure 2, most samples of BE-with-dysplasia images that were misclassified by the attention-based model were predicted as normal tissue. This prediction was likely associated with the BE-with-dysplasia class being the least frequent category in the data set, representing only 11% of the images. For further comparison, see the receiver operating characteristic curves of both models for each class plotted in Figure 3. The attention-based model was trained without ROI annotations yet achieved compelling area under the receiver operating characteristic curve values for each class.

**Figure 4.**
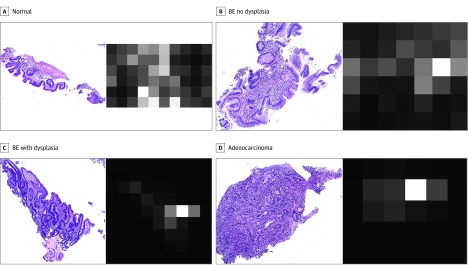
Visualization of Attention Maps Attending Adenocarcinoma Class Features Examples of attention maps are generated by an attention module that is optimized for the features of the adenocarcinoma class. The left column shows whole-slide images from the testing set, whereas the right column shows attention maps of the selected attention module for input images from 4 classes: normal (A), Barrett esophagus (BE) no dysplasia (B), BE with dysplasia (C), and adenocarcinoma (D). Higher attention weight is denoted by white, and lower weight is denoted by black. For visualization purposes, each map is normalized so that its maximum value is 1. The accuracy of attended regions for the adenocarcinoma class images is verified qualitatively by 2 expert pathologists. In contrast, the attention module is inattentive to lower-risk-class images.

The attention maps generated for all of the testing images were visualized to verify the attention mechanism in the proposed model. Characteristic examples for the adenocarcinoma class are presented in Figure 4. The distributions of the attention weights across different classes indicate that the attention module looks for specific features in the adenocarcinoma class (Figure 4D). For images without the target features, the attention weights are low for all regions (Figure 4A and B). In Figure 4C, the attention map is shown to be clinically on target and focused on specific regions in which BE with dysplasia progresses to adenocarcinoma as neoplastic epithelia begin to invade the muscularis mucosae.^46^ eFigure 2 in the Supplement provides more examples.

## Discussion

Results of this diagnostic study demonstrated the ability of attention-based deep learning architecture to detect BE or EAC. The attention-based model’s classification performance on the data set was higher than that of the state-of-the-art sliding window approach. This finding is important because the proposed model needs only reference labels per tissue, whereas the existing sliding window method requires bounding box annotations for each ROI in a tissue. The higher precision in the BE-no-dysplasia and adenocarcinoma classes (ie, lower false-positives in identifying abnormality) achieved by the baseline approach may be associated with heuristic rules developed in consultation with pathologists. The rules, however, were not perfect and thus showed low recall (ie, higher false-negatives). Although both methods used a ResNet-18 model for feature extraction, the attention mechanism of the proposed model further directed the information flow and forced the network to identify local features useful for classification.

The proposed model is directly applicable to high-resolution images without resizing owing to its flexible input design. Because of the time and resources required for annotating microscopy images, having fewer requirements for these annotations would facilitate image analysis research and development. Specifically, tissue-level annotations for training the proposed architecture can potentially be retrieved through searching the pathological reports associated with microscopy images. The proposed model is potentially applicable to histological images of diseases for which training data are scarce or bounding box annotations are not available. To our knowledge, the model is the first to automate the detection of BE and EAC on histopathological slides using a deep learning approach.

### Limitations

This study has some limitations. First, all experiments were conducted on slides collected from a single medical center and scanned with the same equipment. Second, the data set was relatively small compared with conventional data sets in deep learning; in particular, the number of slides of BE with dysplasia was small even after consolidating the classes of BE with low-grade dysplasia and high-grade dysplasia, resulting in lower performance for that class. To evaluate the robustness and generalizability of the proposed model, further verification with different classification tasks and larger data sets from various institutions is required and should be pursued in future research.

Third, even with the proposed method, which was built to analyze entire tissue regions, current graphic processing units do not have enough memory capacity to process very large images. For such slides, we can divide the tissue area into manageable subtissue images. Alternatively, the feature extractor, which is the largest source of memory consumption in the proposed approach, can be optimized to address this issue. The ResNet-18 architecture used in the attention-based model achieved high performance with a relatively low number of parameters. There is, however, room for further reduction of parameters while maintaining high performance, which we intend to pursue in future studies.

## Conclusions

In this diagnostic study, we developed an attention-based model for high-resolution microscopy image analysis. Analogous to how pathologists examine slides under the microscope, the model uses weighted features from the entire slide to classify microscopy images. Results showed that the model marginally outperformed the current sliding window approach on a data set of esophagus tissue with 4 classes (normal, BE-no-dysplasia, BE-with-dysplasia, and adenocarcinoma). Previous methods for analyzing microscopy images were limited by bounding box annotations and unscalable heuristics. The model presented here was trained end to end with labels only at the tissue level, thus removing the need for high-cost data annotation and creating new opportunities for applying deep learning in digital pathology.

## Notes

Supplement. **eMethods 1.** Details of Our Data Annotation Procedure **eMethods 2.** Details of Our Attention-Based Deep Learning Architecture **eFigure 1.** Typical Examples of a Whole-Slide Image and Class-Associated Patches **eFigure 2.** Additional Examples of Visualized Attention Maps Attending to Adenocarcinoma Class Features **eTable.** Class Distribution of Images in Our Dataset **eReferences.**
