# Supplementary material for: Attention-Based Deep Neural Networks for Detection of Cancerous and Precancerous Esophagus Tissue on Histopathological Slides
Source: JAMA Netw Open. 2019 Nov 6;2(11):e1914645. doi: 10.1001/jamanetworkopen.2019.14645 (PMC6865275; doi:10.1001/jamanetworkopen.2019.14645)
Supplement: Supplement. — eMethods 1. Details of Our Data Annotation Procedure eMethods 2. Details of Our Attention-Based Deep Learning Architecture eFigure 1. Typical Examples of a Whole-Slide Image and Class-Associated Patches eFigure 2. Additional Examples of Visualized Attention Maps Attending to Adenocarcinoma Class Features eTable. Class Distribution of Images in Our Dataset eReferences. [file jamanetwopen-2-e1914645-s001.pdf]

Supplementary Online Content

Tomita N, Abdollahi B, Wei J, Ren B, Suriawinata A, Hassanpour S. Attention-based deep neural networks for detection of cancerous and precancerous esophagus tissue on histopathological slides. *JAMA Netw Open.* 2019;2(11):e1914645. doi:10.1001/jamanetworkopen.2019.14645

eMethods 1. Details of Our Data Annotation Procedure

eMethods 2. Details of Our Attention-Based Deep Learning Architecture

eFigure 1. Typical Examples of a Whole-Slide Image and Class-Associated Patches

eFigure 2. Additional Examples of Visualized Attention Maps Attending to Adenocarcinoma Class Features

eTable. Class Distribution of Images in Our Dataset

eReferences.

This supplementary material has been provided by the authors to give readers additional information about their work.

© 2019 Tomita N et al. *JAMA Network Open*

eMethods 1. Details of Our Data Annotation Procedure

In this study, two expert pathologists from the Department of Pathology and Laboratory Medicine at DHMC annotated each whole-slide image by drawing the smallest rectangular bounding boxes around characteristic lesions of each class in each image using Aperio ImageScope software and its Rectangle Tool. The marked ROIs are then extracted as cropped images in JPEG format.

The bounding box annotation is suitable in this study because this method is able to capture the histology patterns without well-defined boundaries, which is suitable for the diagnosis of Barrett's Esophagus based on continuous pathologic patterns. In addition, it reduces the annotation cost on our pathologists. In terms of the costs vs. benefits, a polygon-based annotation is suitable for dense predictions (e.g., a segmentation task), while a bounding box annotation is less demanding and is widely used for classification tasks due to its convenience and robustness.

© 2019 Tomita N et al. *JAMA Network Open*

eMethods 2. Details of Our Attention-Based Deep Learning Architecture

Grid-based Feature Extraction

To extract features on a high-resolution image through a CNN, we first divide the input image into smaller tiles with no overlap (Figure 1a), and then apply a CNN-based feature extraction on each tile (i.e., grid cell) of an r×c grid, with a k feature vector extracted from each cell, resulting in the formation of a structured grid-based feature map U of size k×r×c (Figure 1b). This feature map U is a high-level feature expression of a high-resolution image while preserving the geometric relationships of local features. While the grid-based approach is robust even if full view of a lesion is not in a grid cell due to training with tissue-level geometric augmentation (e.g., random rotation and translation), the granularity of analysis can be further controlled by using overlapping tiles at a higher computational cost. Whereas existing methodology makes a crop prediction solely based on a crop and later aggregates prediction results of crops to build a whole image prediction, our feature structure enables us to directly analyze the whole image through an attention mechanism, which we present in the next subsection below.

In the implementation of CNN architecture for feature extraction, we use the residual neural network (ResNet) architecture,[1] one of the state-of-the-art CNN models with high performance on the ImageNet Large Scale Visual Recognition Competition (ILSVRC) as well as many medical image classification tasks.[2-4] Among several variants of ResNet models, we choose the pre-activation ResNet-18 model.[5] This model achieves a good trade-off between performance and GPU memory usage, which is vital for processing high-resolution images. By removing the final fully-connected layer before the global pooling layer, the network produces a 512-feature vector (k=512) as output for a tile input.

Attention-based Classification

After feature extraction, attention modules are applied to the feature map with their weights determining the importance or value of each tile in diagnostic relevancy (Figure 1c). The importance of each tile is estimated based on features extracted from the tile and its neighboring tiles because the adjoining areas of ROIs can also present informative characteristics. We compute a set of values, $V \in R^{r \times c}$, for a grid. To implement this local valuation function in a deep learning framework while maintaining the robustness for an arbitrary size of grid input, we utilize 3D convolutional filters of size k×d×d, where k corresponds to the size of features and d denotes the height and width of the kernels. In this framework, applying a 3D convolution kernel to a feature map U generates a grid of value estimation V. We normalize V by applying a softmax function to build an attention map α, where i and j are row and column indices:

$$\alpha(V)_{i,j} = \frac{e^{V_{i.j}}}{\sum_{h=1}^{r}\sum_{w=1}^{c} e^{V_{h.w}}} \quad (1)$$

This attention map shows the relative importance of each tile and thus we compute a whole-slide global feature vector using the attention map. Specifically, by treating the attention map α as feature weights, the n-th components of the final feature vector z are computed as follows:

$$z_n = \sum_{h=1}^{r}\sum_{w=1}^{c} \sigma(V)_{h,w} \cdot U_{n,h,w} \quad (2)$$

The feature vector z is subsequently used for whole-slide classification through fully connected layers and a non-linear activation function, allowing for classification of the entire whole-slide image by optimizing for a label.

Moreover, the use of multiple attention modules in our framework can potentially capture more local patterns for classification, increasing the capacity and robustness of the network, especially for medical images of high resolution. As such, we simultaneously apply m 3D filters that generate m attention maps and individually populate m feature vectors. All feature vectors are concatenated to form a single vector, which is fed to the fully connected classifier.

© 2019 Tomita N et al. *JAMA Network Open*

eFigure 1. Typical Examples of a Whole-Slide Image and Class-Associated Patches

(a) A typical whole-slide image in our dataset. This particular slide contains three separate tissues and is of size 9,440 × 15,340 pixels. (b) Samples from each histology class in our dataset.

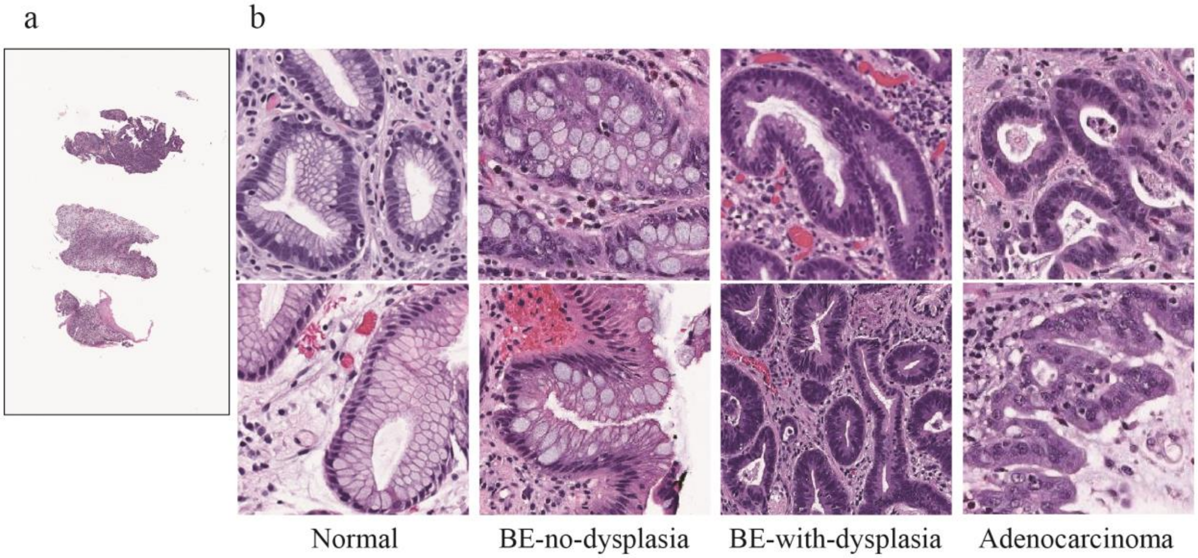

© 2019 Tomita N et al. *JAMA Network Open*

eFigure 2. Additional Examples of Visualized Attention Maps Attending to Adenocarcinoma Class Features

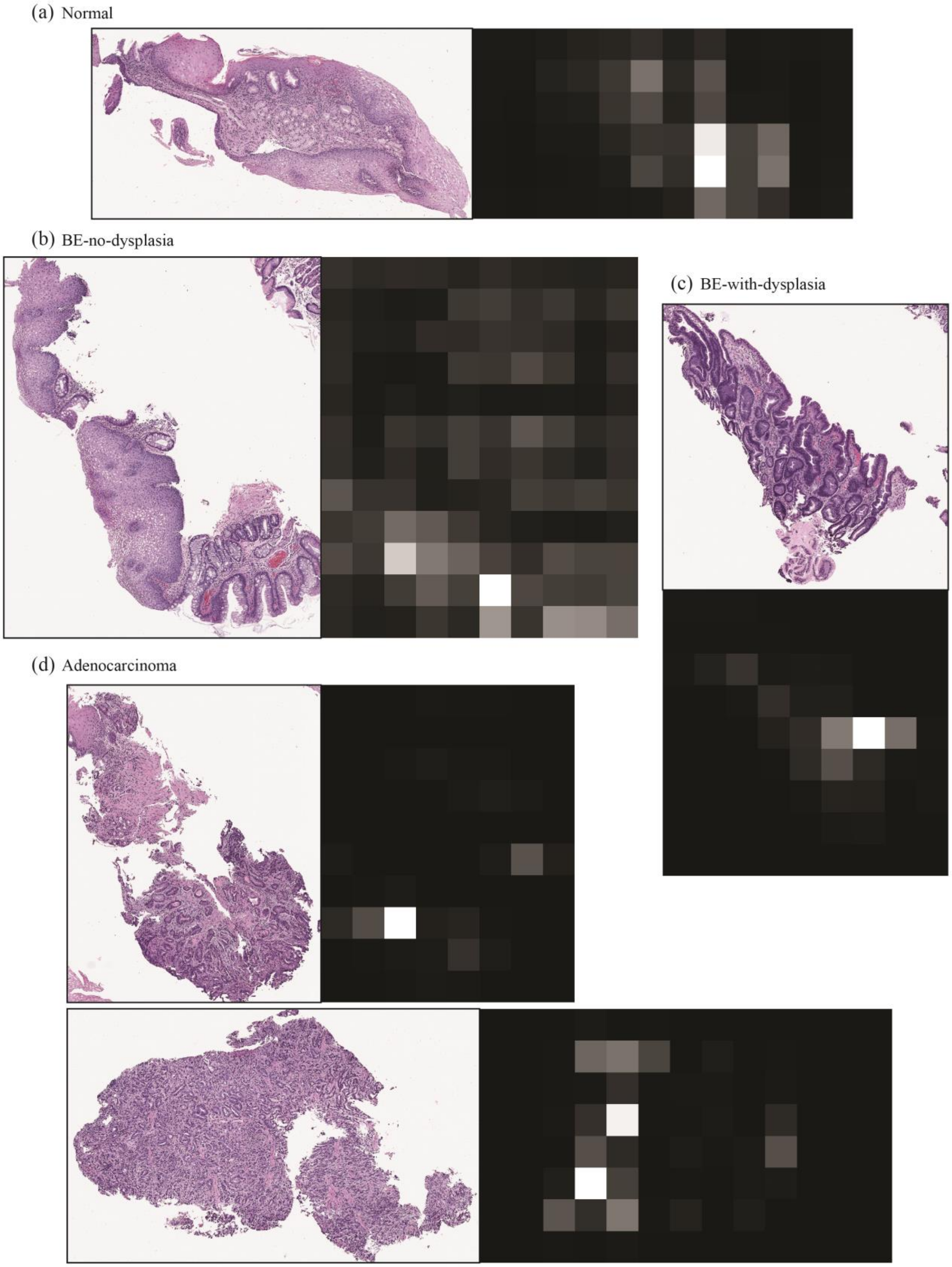

© 2019 Tomita N et al. *JAMA Network Open*

eTable. Class Distribution of Images in Our Dataset

Number (%)			
**Diagnosis**	**Training**	**Validation**	**Test**
Normal	115 (56.1%)	22 (43.1%)	58 (47.2%)
BE-no-dysplasia	37 (18.0%)	13 (25.5%)	30 (24.4%)
BE-with-dysplasia	23 (11.2%)	9 (17.6%)	14 (11.4%)
Adenocarcinoma	30 (14.6%)	7 (13.7%)	21 (17.1%)

© 2019 Tomita N et al. *JAMA Network Open*

eReferences

1. He K, Zhang X, Ren S, Sun J. Deep residual learning for image recognition. *Proceedings of the IEEE conference on computer vision and pattern recognition.* 2016:770-778.
2. Khosravi P, Kazemi E, Imielinski M, Elemento O, Hajirasouliha IJE. Deep convolutional neural networks enable discrimination of heterogeneous digital pathology images. 2018;27:317-328.
3. Chung Y-A, Weng W-HJapa. Learning Deep Representations of Medical Images using Siamese CNNs with Application to Content-Based Image Retrieval. 2017.
4. Zhang Z, Xie Y, Xing F, McGough M, Yang L. Mdnet: A semantically and visually interpretable medical image diagnosis network. *Proceedings of the IEEE conference on computer vision and pattern recognition.* 2017:6428-6436.
5. He K, Zhang X, Ren S, Sun J. Identity mappings in deep residual networks. *European conference on computer vision.* 2016:630-645.

© 2019 Tomita N et al. *JAMA Network Open*
